# Quantitative 3D Evaluation of Facial Soft Tissue Modifications Following Complete Denture Treatment in Edentulous Patients: A Prospective Before–After Study

**DOI:** 10.3390/jcm15020796

**Published:** 2026-01-19

**Authors:** Isabela Toser, Ioana Veja, Adrian Cândea, Andrei-Bogdan Faur, George Dumitru Constantin, Anca-Elena Anghel-Lorinti, Anca Jivănescu

**Affiliations:** 1 Department of Prosthodontics, University of Medicine and Pharmacy Victor Babes, B-dul Revolutiei 1989, No. 9, 300580 Timisoara, Romania; isabela.toser@umft.ro (I.T.); anca.anghel@umft.ro (A.-E.A.-L.); jivanescu.anca@umft.ro (A.J.); 2 Doctoral School, “Victor Babes” University of Medicine and Pharmacy Timisoara, Eftimie Murgu Square 2, 300041 Timisoara, Romania; 3 Department of Dental Medicine, Faculty of Dentistry, “Vasile Goldiș” Western University of Arad, 310025 Arad, Romania; veja.ioana@uvvg.ro; 4 TADERP Research Center, 300041 Timisoara, Romania; 5 Discipline of Clinical Practical Skills, Department I Nursing, Faculty of Medicine, Victor Babeș University of Medicine and Pharmacy, 300041 Timișoara, Romania; george.constantin@umft.ro

**Keywords:** complete denture rehabilitation, 3D facial scanning, edentulous patients, soft tissue morphology, facial anthropometry, digital prosthodontics, facial aesthetics, vertical dimension, nasolabial angle

## Abstract

**Background**: Three-dimensional (3D) facial scanning is an objective, non-invasive method for quantifying facial soft-tissue changes following complete denture (CD) rehabilitation. Reliable quantification of these changes in completely edentulous patients can support more predictable aesthetic and functional outcomes. **Methods**: This prospective before–after observational study included 30 completely edentulous patients (12 men, 18 women; age 48–87 years; mean ± SD: 67.8 ± 9.2 years) who received new maxillary and mandibular CDs. Structured-light 3D facial scans were obtained at baseline (edentulous, without dentures) and post-rehabilitation with dentures in place, in relaxed posture (RP) and maximal intercuspation (MI). Sixty-five validated anthropometric landmarks were analyzed. Primary outcomes were lower facial height (Sn-Gn), nasolabial angle (Cm-Sn-Ls), lower facial convexity (Ls-Li-Pg), mouth width (Ch-Ch), and upper vermilion height (Ls-Sto). Pre–post changes were assessed using paired-sample tests (*p* < 0.05). **Results**: Thirty-four of 65 parameters (52.3%) demonstrated significant post-treatment changes (*p* < 0.05), mainly in the perioral and lower facial regions. The reported parameters were selected due to their clinical relevance in evaluating perioral support and facial profile changes after complete denture treatment. In RP, upper lip thickness increased from 3.69 ± 0.97 mm to 4.96 ± 1.11 mm (Δ = +1.27 mm; *p* < 0.0001) and lower lip thickness from 6.18 ± 2.69 mm to 7.36 ± 1.52 mm (Δ = +1.18 mm; *p* = 0.0408). The nasolabial angle decreased from 116.08 ± 9.17° to 108.06 ± 9.56° (Δ = −8.02°; *p* = 0.0016). In MI, mouth width increased from 55.72 ± 3.43 mm to 57.97 ± 3.13 mm (Δ = +2.25 mm; *p* = 0.0102). **Conclusions**: Complete denture rehabilitation produces measurable, clinically relevant improvements in facial soft-tissue morphology in completely edentulous patients, particularly affecting lip support, mouth width, and the nasolabial profile. Structured-light 3D facial scanning provides a reproducible approach to objective outcome assessment and may support individualized denture design.

## 1. Introduction

Tooth loss leads to substantial aesthetic and functional alterations, significantly disrupting the balance of the facial complex. Progressive alveolar ridge resorption, soft-tissue collapse, and loss of muscular support reduce the vertical dimension of the lower face, alter lip posture, and contribute to a prematurely aged appearance, impaired mastication, and compromised phonetics [1,2,3,4]. These changes are particularly pronounced in completely edentulous individuals, in whom the absence of dentoalveolar structures removes the primary architectural support of the perioral region [5,6]. As a result, diminished lip support and reduced vermilion display often lead to decreased facial expressivity and negatively affect psychosocial well-being, making aesthetic rehabilitation a central objective of prosthodontic treatment [3,7].

Complete dentures (CDs) remain the most widely used therapeutic option for restoring oral function and facial support in edentulous patients. One of the fundamental goals of CD therapy is the reestablishment of an appropriate occlusal vertical dimension (OVD), which plays a critical role in improving lip competence, restoring facial proportions, and rebalancing the spatial relationship between the nose, lips, and chin [8,9]. Despite this importance, clinical evaluation of denture-induced facial changes continues to rely largely on subjective criteria, including phonetic assessment, aesthetic judgement, and tactile feedback. Objective and reproducible documentation of soft-tissue modifications remains limited, and the extent to which complete dentures restore natural facial morphology is still insufficiently quantified.

Traditional facial assessment methods in prosthodontics, such as two-dimensional photography and cephalometric radiography, are constrained by projection errors, limited representation of surface morphology, and restricted reproducibility [10]. In contrast, three-dimensional (3D) facial scanning has emerged as a high-precision, non-invasive imaging modality capable of accurately capturing facial surfaces under natural head position. This technology enables comprehensive, multiplanar analysis of facial soft tissues and supports more objective evaluation of aesthetic outcomes [9,11,12]. Furthermore, the integration of facial scans with intraoral and perioral digital data has strengthened contemporary digital workflows, facilitating more predictable and individualized denture design [13].

Previous studies using 3D facial scanning have shown that complete denture insertion can improve soft-tissue projection, particularly in the lower third of the face. However, most investigations have focused on limited anatomical regions or employed restricted sets of landmarks [6,9]. In addition, few studies have evaluated facial changes across different functional conditions, such as relaxed posture and maximal intercuspation, despite evidence that mandibular positioning and neuromuscular adaptation substantially influence facial contour and soft-tissue behaviour [8]. Consequently, the magnitude, distribution, and clinical relevance of denture-related soft-tissue changes remain incompletely understood.

To address these limitations, the present study focuses exclusively on completely edentulous patients, a population that experiences the most pronounced facial structural deterioration following tooth loss. A comprehensive framework of 65 validated anthropometric landmarks encompassing the upper, middle, and lower facial regions was employed to achieve a detailed characterization of soft-tissue morphology, with particular emphasis on areas highly responsive to denture therapy, such as the perioral and chin regions. Facial measurements were obtained before and after complete denture insertion, both in relaxed posture and maximal intercuspation, allowing evaluation of morphologic changes under distinct yet clinically relevant physiological conditions.

Although previous studies have demonstrated that complete denture insertion influences facial soft-tissue morphology, most investigations have been limited to a small number of landmarks, restricted facial regions, or single static conditions. Moreover, data obtained under different functional mandibular positions, such as relaxed posture and maximal intercuspation, remain scarce, and the clinical relevance of quantified soft-tissue changes is insufficiently documented.

The aim of this prospective before–after study was to quantitatively evaluate facial soft-tissue modifications induced by complete denture rehabilitation using structured-light 3D facial scanning, under both relaxed posture and maximal intercuspation, in completely edentulous patients. By providing objective morphometric data on clinically relevant facial parameters, this study seeks to support evidence-based prosthodontic planning and improve the predictability of aesthetic outcomes in complete denture therapy.

## 2. Materials and Methods

### 2.1. Study Design and Ethical Considerations

A prospective before–after observational study was conducted at the Department of Prosthodontics, Victor Babes University of Medicine and Pharmacy, Timișoara, Romania, between May and July 2025. The study protocol followed the ethical principles outlined in the Declaration of Helsinki and was approved by the institutional Research Ethics Committee (Approval No. 72/20 December 2024). Written informed consent was obtained from all participants prior to enrolment, including consent for the use of clinical data and photo-video documentation.

The primary objective of the study was to quantitatively assess facial soft-tissue modifications induced by complete denture (CD) rehabilitation using structured-light three-dimensional (3D) facial scanning.

### 2.2. Participants

Thirty completely edentulous patients (12 men and 18 women; age range: 48–87 years; mean ± SD: 67.8 ± 9.2 years) were included in the final analysis. All participants presented complete edentulism in both arches and received new maxillary and mandibular complete dentures fabricated at the Department of Prosthodontics, Victor Babes University of Medicine and Pharmacy, Timișoara.

Inclusion criteria were:

- complete edentulism in both arches;
- absence of maxillofacial deformities, visible scars, or dermatological conditions affecting facial contour;
- ability to maintain a natural head position during facial scanning;
- good general health status ensuring reliable facial muscular response, defined as the absence of uncontrolled systemic diseases, neuromuscular disorders, or conditions known to affect facial muscle tone or soft-tissue morphology.

Exclusion criteria were:

- history of maxillofacial trauma or surgical interventions;
- neuromuscular disorders affecting facial expression;
- systemic diseases with potential impact on facial morphology (e.g., uncontrolled diabetes mellitus, connective tissue disorders);
- discomfort, instability, or poor adaptation to the newly delivered dentures during the initial adjustment phase.

All prostheses were fabricated by the same prosthodontic team following standardized clinical and laboratory protocols, using heat-polymerized polymethyl methacrylate (PMMA) denture bases and acrylic resin denture teeth. Occlusal vertical dimension (OVD) and centric relation (CR) were established and verified using conventional phonetic, aesthetic, and functional criteria. Facial scanning was performed 48–72 h after denture delivery to allow initial neuromuscular adaptation and soft-tissue accommodation, while avoiding longer-term remodelling effects.

### 2.3. Three-Dimensional Facial Data Acquisition

Facial morphology was recorded using a structured-light facial scanner (MetiSmile, Shining 3D Tech Co., Ltd., Hangzhou, China; V.2.2.0.X; manufacturer-reported accuracy ±0.1 mm). All scans were acquired in a controlled clinical environment under standardized conditions to minimize variability related to posture, expression, and illumination.

Scanning was performed in a dedicated room with uniform ambient lighting, avoiding direct light sources or shadows on the facial surface. A neutral, non-reflective background was used. The scanner was mounted on a fixed support, and the working distance between the scanner and the patient was maintained at approximately 500 mm, in accordance with the manufacturer’s recommendations.

Patients were seated upright on a rotating chair and positioned in natural head position (NHP). NHP was achieved by instructing participants to look straight ahead at a fixed point at eye level placed on the opposite wall, without external head support. Head position was visually checked by the operator before each acquisition. No head restraints were used.

To standardize facial expression, participants were instructed to keep the lips gently closed at rest, without smiling, clenching, or pursing, and to avoid speaking, swallowing, or facial movements during scanning. For scans acquired in maximal intercuspation (MI), patients were instructed to occlude lightly in centric occlusion without excessive muscular contraction.

**Figure 1:**
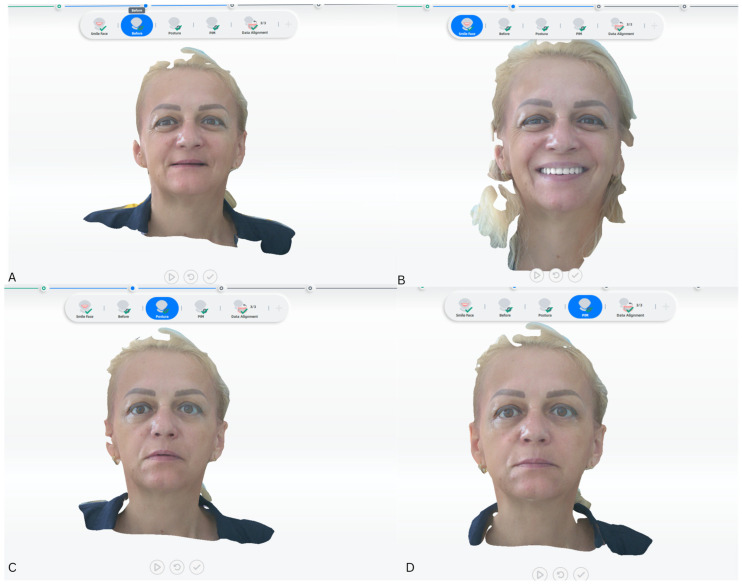
Standardized three-dimensional (3D) facial scanning conditions applied in the study. (**A**) Baseline facial scan performed in the edentulous condition, without dentures. (**B**) Post-rehabilitation facial scan with complete dentures inserted during smiling, acquired to document dynamic perioral soft-tissue behaviour. (**C**) Post-rehabilitation facial scan with complete dentures inserted in relaxed posture (RP). (**D**) Post-rehabilitation facial scan with complete dentures inserted in maximal intercuspation (MI).

Each participant underwent one scan per condition (Figure 1). No repeated captures were performed unless a scan was visibly affected by motion artefacts or incomplete surface acquisition, in which case the scan was immediately repeated. The final dataset therefore consisted of one high-quality scan per condition for each participant.

Facial surface data were exported from the MetiSmile software (V.2.2.0.X) in standard triangulated mesh format (STL). The default software export settings were used, preserving the native mesh density generated by the scanner; no additional smoothing or mesh decimation was applied prior to analysis.

All facial models were automatically aligned using the software’s best-fit registration algorithm. To ensure consistency and to minimize distortion related to soft-tissue displacement, alignment was performed using relatively stable facial regions, including the forehead, nasal bridge, and upper nasal dorsum. Mobile regions of the lower face and perioral area were excluded from the alignment process. This approach was applied consistently across all scanning conditions (baseline, RP, and MI).

All scans were acquired by the same experienced operator following an identical protocol to reduce operator-dependent variability.

### 2.4. Landmark Identification and Measurement Protocol

A total of 65 validated three-dimensional anthropometric landmarks were used to characterize facial soft-tissue morphology. The complete list of landmarks, together with their anatomical definitions and abbreviations, is provided in Supplementary Materials, in accordance with established craniofacial anthropometric and 3D morphometric guidelines.

Landmark identification was performed using the proprietary MetiSmile software on aligned facial surface models. All landmarks were manually placed and verified by a single examiner with prior experience in 3D facial morphometric analysis. Before the start of the study, the examiner underwent calibration through repeated landmark placement on a set of training scans not included in the final analysis, to ensure consistency in landmark interpretation and positioning.

The examiner was not blinded to the scanning condition (baseline versus post-rehabilitation), as the presence or absence of dentures was visually evident on the facial models. This limitation is acknowledged; however, a standardized landmark definition protocol was strictly applied across all scans to minimize systematic bias.

To assess intra-operator reliability, landmark placement and subsequent measurements were repeated in 10 randomly selected participants after a one-week interval, under identical conditions. Reliability was quantified using intraclass correlation coefficients (ICC) calculated with a two-way mixed-effects model for absolute agreement (ICC 3,1), which is appropriate when measurements are performed by a single fixed examiner.

ICC values exceeded 0.90 for all linear and angular variables, indicating excellent intra-operator reliability and confirming the robustness of the landmark identification and measurement protocol.

**Figure 2:**
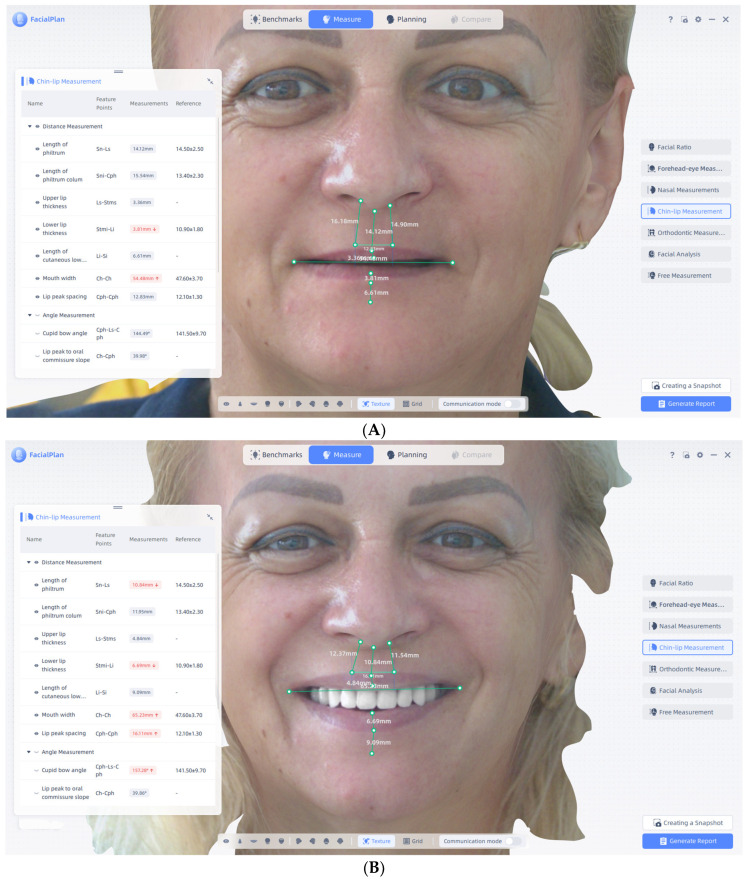

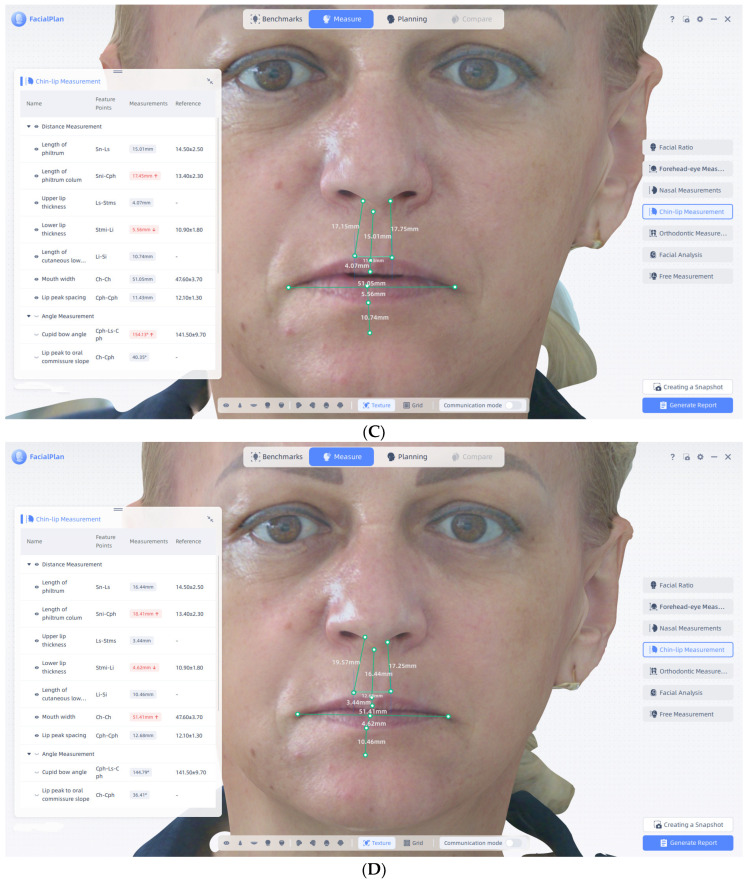
Representative visualization of anthropometric landmark identification and facial soft-tissue measurements across the four scanning conditions. (**A**) Baseline edentulous scan illustrating the distribution of validated facial landmarks and example linear and angular measurements. (**B**) Post-rehabilitation smiling scan with landmarks displayed to illustrate dynamic soft-tissue displacement (qualitative reference). (**C**) Post-rehabilitation relaxed posture (RP) scan showing landmark placement and representative measurements used for quantitative analysis. (**D**) Post-rehabilitation maximal intercuspation (MI) scan demonstrating landmark positioning and measurement construction under functional mandibular stabilization.

The same landmark definitions and measurement protocol were consistently applied across all scanning conditions (Figure 2). RP and MI scans were used for quantitative analysis, while smiling scans served illustrative purposes only.

### 2.5. Primary Measurement Outcomes

Outcome measures were selected based on their established clinical relevance in the evaluation of facial morphology in edentulous patients:

- Subnasale-Gnathion (Sn-Gn): vertical lower facial height;
- Nasolabial angle (Cm-Sn-Ls);
- Lower facial convexity angle (Ls-Li-Pg);
- Intercommissural distance (Ch-Ch): mouth width;
- Upper vermilion height (Ls-Sto).

All measurements were compared between baseline and post-rehabilitation scans, with post-treatment evaluations conducted in both relaxed posture (RP) and maximal intercuspation (MI).

### 2.6. Statistical Analysis

Statistical analyses were performed using MedCalc (v23.4.0, Ostend, Belgium). Data normality was assessed using the Shapiro–Wilk test. Pre–post comparisons were performed using paired-sample *t*-tests for normally distributed variables and the Wilcoxon signed-rank test otherwise; *p* < 0.05 was considered significant. Primary outcomes were predefined based on clinical relevance, while the remaining variables were analyzed exploratorily. Given the exploratory scope and the limited number of predefined primary outcomes, no formal correction for multiple comparisons was applied to avoid inflating type II error; therefore, *p*-values for exploratory outcomes were interpreted cautiously and descriptively rather than as confirmatory evidence. No formal sample size or power calculation was performed.

Based on their clinical relevance in prosthodontics, the following parameters were defined as primary outcomes: vertical lower facial height (Sn–Gn), nasolabial angle (Cm–Sn–Ls), lower facial convexity angle (Ls–Li–Pg), intercommissural width (Ch–Ch), and upper vermilion height (Ls–Sto). All remaining variables were treated as exploratory outcomes and were analyzed to provide complementary descriptive information regarding overall facial soft-tissue modifications.

## 3. Results

### 3.1. Descriptive Analysis

Thirty completely edentulous patients (12 men, 18 women; mean age 67.8 ± 9.2 years) successfully completed the 3D facial scanning procedures both at baseline and after complete denture insertion. No adverse reactions or scanning difficulties were observed. Intra-operator reliability demonstrated excellent reproducibility for all variables assessed, with ICC values exceeding 0.90 for both linear (mm) and angular (°) measurements, confirming the robustness of landmark identification and the analytical workflow.

### 3.2. Overall Changes in Facial Landmark Measurements

Among the 65 analyzed parameters, 34 showed statistically significant changes (*p* < 0.05), including the predefined primary outcomes; the remaining significant variables were analyzed exploratorily. The significant changes were predominantly observed in the circumoral and lower facial regions, which are most directly influenced by denture-mediated restoration of dentoalveolar support. These modifications were associated with enhanced lip volume, improved soft-tissue projection, and increased perioral tension. Changes were evident in both relaxed posture (RP) and maximal intercuspation (MI), with generally greater effects observed in MI, suggesting improved neuromuscular engagement following occlusal stabilization.

### 3.3. Linear Measurements

Table 1 summarizes MI outcomes. A statistically significant increase in mouth width was recorded (+2.25 mm, *p* = 0.0102), indicating improved lateral expansion of the perioral envelope and re-establishment of proper lip stretch over the denture flanges. Although lip thickness values increased slightly in MI, these variations did not reach statistical significance (*p* > 0.05), suggesting that lip tension improves primarily during functional mandibular support rather than static positioning.

In RP (Table 2), upper lip thickness demonstrated a highly significant increase of +1.27 mm (*p* < 0.0001), while lower lip thickness increased by +1.18 mm (*p* = 0.0408). These findings reflect restoration of anterior support for the upper and lower labial soft tissues, which are notably compromised in edentulous ageing patients. Mentolabial sulcus depth showed a mild increase post-rehabilitation but did not reach significance, indicating that deeper profile changes at the chin level may require longer adaptation or different prosthetic designs.

Taken together, these results suggest that the reestablished dentoalveolar framework contributes to improved labial tonicity and natural soft-tissue projection.

### 3.4. Angular Measurements

Angular analysis demonstrated post-rehabilitation profile refinement, especially in RP. The nasolabial angle decreased significantly (−8.02°, *p* = 0.0016), indicating anterior support of the upper lip and a more favourable relationship between the nasal base and lip line. This change is clinically meaningful, as increased nasolabial angle is a known marker of facial ageing and perioral collapse in edentulous individuals.

In MI, the nasolabial angle also showed a decreasing trend (−2.59°), but the difference did not reach statistical significance (*p* = 0.2629), likely due to greater inter-individual variability under functional mandibular engagement. Mentolabial angle changes were minimal and statistically insignificant in both positions, which aligns with the modest linear modifications observed in the chin-labial region.

### 3.5. Clinical Relevance and Esthetic Impact

Post-rehabilitation modifications suggest that complete dentures effectively:

- restore key perioral volume deficiencies;
- increase soft-tissue tension and functional support in the lower third of the face;
- improve profile harmony and aesthetic appeal, particularly at the nasolabial level;
- improve overall facial proportions.

Enhancements were consistently greater in MI, reinforcing the importance of optimal occlusal stabilization for soft-tissue performance. The observed changes—though varying in magnitude across individuals—demonstrate that complete dentures do more than restore dentition; they contribute to measurable modifications of the external facial envelope in a clinically relevant manner.

## 4. Discussion

This study demonstrated that complete denture rehabilitation is associated with measurable and clinically relevant modifications of facial soft-tissue morphology, predominantly affecting the lower third of the face. A substantial proportion of the analyzed anthropometric landmarks exhibited statistically significant post-treatment changes, indicating that complete dentures contribute not only to the restoration of intraoral function but also to the structural support of compromised perioral soft tissues [13,14,15]. Increases in upper and lower lip thickness, particularly in relaxed posture, reflect re-establishment of anterior dentoalveolar support, which is progressively lost following tooth extraction and alveolar ridge resorption [16,17]. In maximal intercuspation, the observed increase in mouth width suggests improved circumoral soft-tissue tension and enhanced prosthesis stability under functional loading conditions [18]. In relaxed posture, the observed increases in upper and lower lip thickness and the reduction in the nasolabial angle are consistent with previous 3D facial scanning studies reporting improved anterior soft-tissue support following complete denture insertion. In maximal intercuspation, the greater changes in mouth width and perioral tension suggest enhanced neuromuscular engagement under occlusal stabilization, supporting findings from earlier functional facial assessments in prosthodontic rehabilitation.

Among angular parameters, the reduction in the nasolabial angle represents a clinically relevant aesthetic outcome. In edentulous patients, this angle commonly becomes more obtuse because of upper lip retrusion and reduced vertical dimension; therefore, its partial normalization following denture insertion is consistent with restoration of anterior support and improved facial proportions [13,15,19]. In contrast, changes in mentolabial sulcus morphology were limited and did not reach statistical significance, which may be attributed to long-standing skeletal atrophy and reduced soft-tissue elasticity in the lower lip–chin complex—features that are often insufficiently compensated by conventional complete dentures alone [20,21].

Compared with dentate individuals or patients rehabilitated with implant-supported prostheses, the magnitude of soft-tissue changes observed after complete denture therapy appears more pronounced in the perioral and lower facial regions, reflecting the greater baseline loss of dentoalveolar support in completely edentulous patients. While direct comparisons were not performed, this contextualization helps interpret the extent of the observed morphologic changes.

The magnitude of several morphologic changes observed in the present investigation is likely to be clinically perceptible, based on previously reported ranges of visually detectable differences in facial aesthetics. Prior studies suggest that changes on the order of approximately 1 mm in labial projection, around 2 mm in mouth width, or reductions of 5° or more in the nasolabial angle may be perceived by clinicians and lay observers as aesthetically meaningful [22,23,24]. While these values do not represent universally standardized minimal important morphologic difference (MINMD) thresholds, multiple parameters in the current analysis approached or exceeded these ranges, supporting the clinical relevance of the findings beyond statistical significance. These results align with established prosthodontic principles indicating that restoration of occlusal vertical dimension contributes to improved facial proportions, lip competence, and overall facial harmony [11,19]. Although changes exceeding approximately 1 mm or 5° are often considered visually perceptible, it should be noted that minimal important morphologic difference thresholds are not standardized and may vary depending on facial region, observer sensitivity, and assessment method.

Three-dimensional facial scanning played a central role in identifying these outcomes, offering high spatial accuracy and eliminating projection-related limitations inherent to two-dimensional imaging methods [9,12,25]. Assessment of facial morphology in both relaxed posture and maximal intercuspation enabled differentiation between static soft-tissue configuration and functionally stabilized conditions, providing insight into the influence of occlusal support and neuromuscular engagement on facial soft-tissue behaviour.

Several limitations should be considered when interpreting these findings. Facial scans were acquired 48–72 h after denture insertion, a timeframe selected to allow initial neuromuscular accommodation while minimizing fatigue; however, this interval does not reflect medium- or long-term adaptation of facial soft tissues, which may continue to evolve with prolonged denture wear. Additionally, although a smiling scan was obtained to document dynamic perioral behaviour, it was included for qualitative illustration only and excluded from quantitative analysis due to the inherent variability of facial expressions. The lack of a dentate or implant-supported control group further limits direct comparison with alternative rehabilitation strategies. Although all scans were acquired and analyzed by a single experienced operator and reproducibility was excellent (ICC > 0.90), minor alignment deviations between scans cannot be fully excluded. Finally, the sample size restricts extrapolation to patients with more advanced facial collapse or different skeletal patterns. Another limitation of the present study is the absence of symmetry analysis and volumetric assessment of soft-tissue changes, which could provide additional insight into three-dimensional facial adaptation following complete denture rehabilitation.

Future research should focus on the long-term stability of these soft tissue changes and on whether additional morphologic adaptation occurs over time. Integration of patient-reported outcome measures related to aesthetic satisfaction, psychosocial impact, and functional comfort would further strengthen the clinical interpretation of morphometric data. Moreover, dynamic 3D analysis, volumetric deviation mapping, and artificial intelligence-assisted methods may provide a more comprehensive understanding of facial soft-tissue responses to prosthetic rehabilitation [26]. Future studies should include longitudinal assessments at standardized follow-up intervals, such as 6 and 12 months after denture delivery, to evaluate the stability and progression of facial soft-tissue adaptations over time.

## 5. Conclusions

Complete denture rehabilitation was associated with measurable improvements in facial soft-tissue morphology, particularly within the lower third of the face. Quantitative 3D analysis showed enhanced perioral support, increased lip volume, increased mouth width, and a more favourable nasolabial profile, consistent with restoration of dentoalveolar support and soft-tissue tension. Several changes were of a magnitude likely to be perceived as clinically relevant, based on prior facial perception studies. Three-dimensional facial scanning proved to be a reliable and objective method for documenting prosthetic outcomes, offering superior accuracy compared with traditional visual or two-dimensional assessments.

## Figures and Tables

**Table 1:** Linear and angular soft-tissue measurements in maximal intercuspation (MI).

Parameter	Before (MI) Mean ± SD	After (MI) Mean ± SD	Mean Δ	Unit	*p*-Value	95% CI
Upper lip thickness	4.82 ± 0.95	4.40 ± 1.08	−0.42	mm	0.1152	−0.946 to 0.106
Lower lip thickness	6.66 ± 1.37	6.47 ± 1.56	−0.19	mm	0.6181	−0.949 to 0.569
Mouth width (Ch-Ch)	55.72 ± 3.43	57.97 ± 3.13	+2.25	mm	0.0102	0.553 to 3.947
Nasolabial angle (Cm-Sn-Ls)	109.27 ± 9.10	106.68 ± 8.64	−2.59	°	0.2629	−7.176 to 1.996
Mentolabial sulcus depth	2.67 ± 1.17	2.58 ± 1.28	−0.09	mm	0.7772	−0.724 to 0.544

SD = standard deviation. Statistical significance was interpreted alongside the magnitude of change to assess potential clinical relevance.

**Table 2:** Linear and angular soft-tissue measurements in relaxed posture (RP).

Parameter	Before (RP) Mean ± SD	After (RP) Mean ± SD	Mean Δ	Unit	*p*-Value	95% CI
Upper lip thickness	3.69 ± 0.97	4.96 ± 1.11	+1.27	mm	<0.0001	0.731 to 1.809
Lower lip thickness	6.18 ± 2.69	7.36 ± 1.52	+1.18	mm	0.0408	0.0508 to 2.309
Mouth width (Ch-Ch)	61.68 ± 4.25	63.40 ± 3.76	+1.72	mm	0.1023	−0.354 to 3.794
Nasolabial angle (Cm-Sn-Ls)	116.08 ± 9.17	108.06 ± 9.56	−8.02	°	0.0016	−12.861 to −3.179
Mentolabial sulcus depth	2.09 ± 0.89	2.50 ± 1.05	+0.41	mm	0.1082	−0.093 to 0.913

Statistical significance was interpreted alongside the magnitude of change to assess potential clinical relevance.

## Data Availability

Data are available from the corresponding authors upon reasonable request.

## Supplementary Materials

The following supporting information can be downloaded at: https://www.mdpi.com/article/10.3390/jcm15020796/s1.

## Abbreviations

The following abbreviations are used in this manuscript:

OVD	Occlusal Vertical Dimension
MI	Maximal Intercuspation
ICC	Intraclass Correlation Coefficient
3D	Three-Dimensional
CD	Complete Denture

## References

[1] Shaw R.B., Katzel E.B., Koltz P.F., Kahn D.M., Girotto J.A., Langstein H.N. (2010). Aging of the mandible and its aesthetic implications. Plast. Reconstr. Surg..

[2] Sforza C., Grandi G., Binelli M., Tommasi D.G., Rosati R., Ferrario V.F. (2009). Age- and sex-related changes in the normal human ear. Forensic Sci. Int..

[3] Coleman S.R., Grover R. (2006). The Anatomy of the Aging Face: Volume Loss and Changes in 3-Dimensional Topography. Aesthetic Surg. J..

[4] Hernández E.L., Alvarez A., Abou-Ayash S., Att W. (2022). Effect of Complete Dentures on Facial Soft Tissue Volume: A 3D Comparative Study. Int. J. Prosthodont..

[5] Sharma P., Arora A., Valiathan A. (2014). Age changes of jaws and soft tissue profile. Sci. World J..

[6] Sarver D.M., Ackerman M.B. (2003). Dynamic smile visualization and quantification: Part 1. Evolution of the concept and dynamic records for smile capture. Am. J. Orthod. Dentofac. Orthop..

[7] Niwatcharoenchaikul W., Tumrasvin W., Arksornnukit M. (2014). Effect of complete denture occlusal schemes on masticatory performance and maximum occlusal force. J. Prosthet. Dent..

[8] Lo Russo L., Di Gioia C., Salamini A., Guida L. (2020). Integrating intraoral, perioral, and facial scans into the design of digital dentures. J. Prosthet. Dent..

[9] Jivănescu A., Bratu D.C., Tomescu L., Măroiu A.C., Popa G., Bratu E.A. (2015). The assessment of lower face morphology changes in edentulous patients after prosthodontic rehabilitation, using two methods of measurement. Rom. J. Morphol. Embryol..

[10] Carlsson G.E., Omar R. (2010). The future of complete dentures in oral rehabilitation. A critical review. J. Oral Rehabil..

[11] Little A.C., Jones B.C., DeBruine L.M. (2011). Facial attractiveness: Evolutionary based research. Philos. Trans. R. Soc. B: Biol. Sci..

[12] Hajaj T., Marian D., Zaharia C., Niculescu S.T., Negru R.M., Titihazan F., Rominu M., Sinescu C., Novac A.C., Dobrota G. (2025). Fracture Resistance of CAD/CAM-Fabricated Zirconia and Lithium Disilicate Crowns with Different Margin Designs: Implications for Digital Dentistry. J. Funct. Biomater..

[13] Toyoshima G.H.d.L., Pucciarelli M.G.R., Neppelenbroek K.H., Sforza C., de Menezes M., Oliveira T.M., Soares S. (2021). Evaluation by 3D stereophotogrammetry of facial changes in edentulous patients after rehabilitation. J. Appl. Oral Sci. Rev. FOB.

[14] Lefrançois E., Delanoue V., Morice S., Ravalec X., Desclos-Theveniau M. (2025). A Digital Approach for a Complete Rehabilitation with Fixed and Removable Prostheses: A Technical Procedure. Dent. J..

[15] Skomina Z., Kuhar M., Verdenik M., Hren N.I. (2025). Stereophotometric facial changes in edentulous older adults after rehabilitation with complete dentures. Gerodontology.

[16] Demirekin Z.B., Gunaydin A., Cavdarli K., Findik Y., Turkaslan S., Baykul T. (2022). 3D assessment of facial contours of patients wearing either complete denture or implant-supported fixed dentures. Niger. J. Clin. Pract..

[17] Hassan B., Tahmaseb A., Jacobs R., Bornstein M.M. (2014). Three-dimensional Facial Scanning Technology: Applications and Future Trends. Forum Implantol..

[18] Chisnoiu A.M., Chira O., Marginean I., Iacob S., Hrab D., Păstrav O., Fluerașu M., Chisnoiu R.M., Păstrav M. (2025). How to Be Predictable in the Management of Vertical Dimension of Occlusion-A Narrative Review and Case Report. Oral.

[19] Ceylan G., Özel G.S., Memişoglu G., Emir F., Şen S. (2023). Evaluating the Facial Esthetic Outcomes of Digital Smile Designs Generated by Artificial Intelligence and Dental Professionals. Appl. Sci..

[20] Benhamida S.A., El Maroush M.A., Elgendy A.A., Elsaltani M.H. (2019). Residual ridge resorption, the effect on prosthodontics management of edentulous patient: An article review. Int. J. Sci. Res. Manag..

[21] Xiao Y., Mao B., Nie J., Liu J., Wang S., Liu D., Zhou Y. (2024). Accuracy Evaluation of a Three-Dimensional Face Reconstruction Model Based on the Hifi3D Face Model and Clinical Two-Dimensional Images. Bioengineering.

[22] Alrbata R.H., Alfaqih A.K., Almhaidat M.R., Al-Tarawneh A.M. (2020). Thresholds of Abnormality Perception in Facial Esthetics among Laypersons and Dental Professionals: Frontal Esthetics. Int. J. Dent..

[23] Strauss F.J., Gil A., Smirani R., Rodriguez A., Jung R., Thoma D. (2024). The use of digital technologies in peri-implant soft tissue augmentation—A narrative review on planning, measurements, monitoring and aesthetics. Clin. Oral Implant. Res..

[24] Major M., Mészáros B., Würsching T., Polyák M., Kammerhofer G., Németh Z., Szabó G., Nagy K. (2024). Evaluation of a Structured Light Scanner for 3D Facial Imaging: A Comparative Study with Direct Anthropometry. Sensors.

[25] Schendel S. (1995). Anthropometry of the Head and Face. Plast. Reconstr. Surg..

[26] Hajaj T., Lile I.E., Veja I., Titihazan F., Rominu M., Negruțiu M.L., Sinescu C., Novac A.C., Talpos Niculescu S., Zaharia C. (2025). Influence of Pontic Length on the Structural Integrity of Zirconia Fixed Partial Dentures (FPDs). J. Funct. Biomater..

